# Development and validation of a questionnaire to measure the severity of pain, functional limitations, and reduction of sports ability for german-speaking patients with osteochondral lesions of the ankle (OCLA-G)

**DOI:** 10.1186/s12891-023-06445-3

**Published:** 2023-05-01

**Authors:** Heinz Lohrer, Stephanie Wagner, Markus Wenning, Jan Kühle, Hagen Schmal, Albert Gollhofer

**Affiliations:** 1 Department for Sports and Sport Science, University of Freiburg, Schwarzwaldstraße 175, Freiburg, 79117 Germany; 2 European SportsCare Network (ESN), Zentrum für Sportorthopädie, Borsigstrasse 2, Wiesbaden-Nordenstadt, 65205 Germany; 3 BDH Klinik Waldkirch gGmbH, Heitere Weg 10, Waldkirch, 79183 Germany; 4 Department of Orthopedics and Trauma Surgery, Medical Center, University of Freiburg, Hugstetter Straße 55, Freiburg, 79106 Germany

**Keywords:** Osteochondral lesion, Ankle, Patient-reported outcomes measures, Psychometrics, Validity

## Abstract

**Background:**

There is no patient-reported functional scale specific for osteochondral lesion of the ankle (OCLA). Therefore, the objectives of this study were to develop a questionnaire that measures symptom severity, function, and sports capacity in patients with osteochondral lesions of the ankle and to determine the psychometric properties of the tool in German language (OCLA-G).

**Methods:**

The OCLA-G questionnaire was developed according to the COSMIN guidelines. Scalable items were generated from a literature search, based on an evaluation of 71 own OCLA patients, and from expert opinions. Following a twofold item reduction the questionnaire underwent explorative data analysis and principal component analysis. Validity and reliability were analysed in four groups of participants (40 patients with OCLA, 40 patients with other foot and ankle injuries, 40 asymptomatic athletes serving as a population at risk, and 40 asymptomatic persons playing sports not at risk). The minimum age for participation in the study was set at 18 years. The mean age was 39.3 ± 15.1 years.

**Results:**

The final OCLA-G questionnaire consists of eight and five questions to mirror activities of daily life (ADL) and sports, respectively. Excellent internal consistency (Cronbach’s α = 0.950 for the ADL subscore and 0.965 for the sport subscale, respectively) was found. Spearman’s rank correlation coefficients for test-retest reliability were 0.992 for the ADL subscore and 0.999 for the sport subscale (p < 0.001). The results of the exploratory and confirmatory factor analyses indicated that item difficulty was between 23.4 and 62.8. The Pearson correlation for the OCLA subscales ADL and sport was 0.853 (p < 0.001). Construct validity as tested against the SF-12 questionnaire subscales (Physical and Mental component scale) were r = -0.164 to -0.663 (p < 0.05). Statistically, there was no ADL and sport OCLA mean score difference between OCLA patients and patients with other foot and ankle injuries (p = 0.993 and 0.179, respectively), but both groups differed from the uninjured control groups (p < 0.001). There were no ceiling or floor effects.

**Conclusions:**

The OCLA-G was successfully developed as the first patient reported and injury specific outcome scale to measure the impact of OCLA induced symptoms on activities of daily living and sport. This study provides evidence for the reliability and validity of the OCLA-G assessing patients with OCLA.

**Trial registration:**

The registration trial number is DRKS00009401 on DRKS. ‘Retrospectively registered’. Date of registration: 10/12/2015.

## Background

Osteochondral lesions are defined as joint injuries, involving both cartilage and subchondral bone, regardless of its cause [1]. Osteochondral lesions of the ankle (OCLA) can occur to the joint forming areas of the distal tibia, the talus, and the malleoli. Genetic, metabolic, and endocrine causes are still discussed, but traumatic or microtraumatic origin seems most likely [1]. The main feature of OCLA is pain after a traumatic event or chronic ankle pain, but further unspecific symptoms like swelling, stiffness, and weakness may be present [1]. Less frequently but more specifically, patients present locking phenomena. Symptoms are typically triggered by load during daily life and affect the physical performance of both recreational and elite athletes.

The results of manual examinations and imaging do not necessarily correspond to the patient’s self-assessment. Therefore, the subjectively perceived symptoms (patient satisfaction) and their functional effects are key criteria for the assessment of the severity, for therapy, monitoring, and to assess the outcome in studies [2].

In the literature, there is a reported clinical measurement’s deficiency for OCLA and 46 different instruments were used so far [3]. Besides the fact that the outcome is not comparable between the respective investigations when measured with 46 different instruments, condition-specific PROMs provide more clinical details, related directly to the investigated condition. As a result, the clinician receives more useful information to better address the particular condition in the future. In general, condition-specific Patient Reported Outcome Measures (PROMs) provide advantages over generic PROMs due to their higher sensitivity to change, e.g., to document the „benefits of an intervention” in the target population [4, 5]. To objectively quantify the severity of symptoms and loss of function associated with OCLA, generic patient-reported outcome measures (PROMs) have been used. Literature analysis revealed no validated, condition-specific, and patient-administered instrument to determine the severity of symptoms, functional impairment, and ability to play sports for patients with OCLA [3].

The purpose of this study was to develop a patient-oriented and disease-specific questionnaire for German speaking patients with OCLA and to evaluate its psychometric properties. The OCLA-G (G stands for German language) questionnaire intends to measure pain-related and functional limitations during activities of daily living and sports.

## Methods

The development of the OCLA-G questionnaire followed the COnsensus-based Standards for the selection of health status Measurement INstruments (COSMIN) recommendations [6].

We conducted the study according to the revised Declaration of Helsinki by The World Medical Association [7].

The Ethics Committee (Ethik-Kommission der Albert-Ludwigs-Universität Freiburg, Germany) approved the study. The application number is 366/15.

Written informed consent was obtained from all the patients/participants willing to participate in the study. The rights of the patients and control participants were protected. The registration trial number is DRKS00009401 on DRKS. ‘Retrospectively registered’. Date of registration: 10/12/2015. The study was conducted between 07/01/2018 and 04/30/2021.

### Phase 1: development of the OCLA-G questionnaire

#### Expert groups

Two expert panels were formed to develop the OCLA-G questionnaire. The first expert group (EG1) consisted of an orthopaedic surgeon, specialized in sports and exercise medicine and physical therapy and rehabilitation (HL), a sports scientist specialized in clinical research and development, cross-cultural translation, and validation of questionnaires (TN) and a medical doctor in residency training for orthopaedics and trauma surgery (SW). A second expert panel (EG2) additionally consisted of a sports scientist specialized in biomechanics (AG), an orthopaedic surgeon serving also as contact person for the “Module Ankle Joint” of the Cartilage-Register within the Deutsche Gesellschaft für Orthopädie und Unfallchirurgie (DGOU) (MA), another orthopaedic surgeon serving as contact person for the “Module Knee Joint” of the Cartilage-Register DGOU (PN). Two sports physiotherapists (BH, LM), and an OCLA patient with a medical background (PM) completed the EG 2 panel.

#### Item generation

**Fig. 1:**
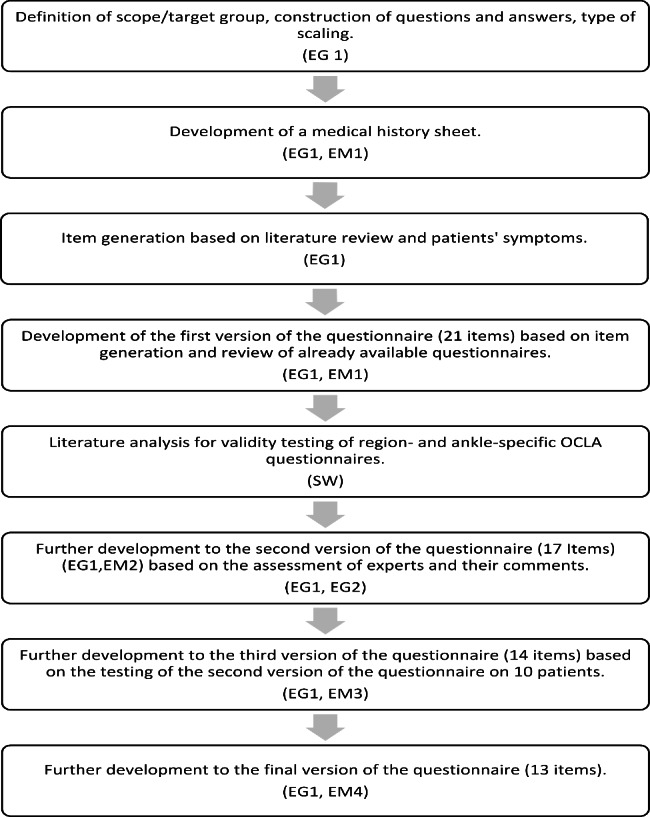
Flow-chart demonstrating the stepwise development and related expert meetings (EM) of the different expert groups (EG 1, EG 2)

The questionnaire was developed in a stepwise procedure [8–10] (Fig. 1). Initially, the purpose of the questionnaire was determined and the scope, i.e. the target group was defined. In addition, a medical history sheet was developed to obtain information about anthropometric data, additional injuries, accompanying diseases, duration of the symptoms, ability to work, family predisposition, triggering events or previous trauma, current athletic activities, or changed athletic activity due to OCLA. In addition, questions on the previous imaging procedures, the diagnosed degree of OCLA, and the respective treatments were included.

The construction of questions, their possible answers, and the scalability were discussed by the experts on the basis of the existing literature. For this purpose, a literature review was conducted to screen for validity of existing region- and ankle-specific questionnaires. This was followed by item generation, construction of the questionnaire, and a twofold item reduction. Expert group 1 developed a first version of the questionnaire. Four members of the expert committees independently rated the version 1 (V1) items for relevance of the questions using a Likert box with six response options ranging from “very useful” (6 points) to “not relevant to OCLA” (1 point). A high column total indicated a meaningful respective item. The results were analysed and written comments of all experts were objected to consensus discussion in another EG 1 meeting. Following item reduction, a pretest of this version 2 (V2) was performed on ten OCLA patients. These OCLA patients were selected from the recent electronic charts of the orthopedic department of the Institute for Sports Medicine, Frankfurt am Main/Germany. The patients filled out the questionnaire and were asked to address any incomprehensibility or problems they had in answering or handling the questionnaire. All ten patients received written information and explanations about the questionnaire and the study. The time needed to complete the questionnaire was recorded in a pilot application. After completion of the questionnaire practical characteristics (missing responses, readability, and comprehensibility) were discussed between the researcher and the patients. The ten completed V2 questionnaires were evaluated for the sum of the answers, their mean value and for the difficulty index Pi for each item. To develop the prefinal questionnaire (V3), the results were analysed by consensus discussion in another EG 1 meeting, and this version 3 underwent further formal validation.

The prefinal questionnaire was subjected to exploratory data analysis, principal component analysis, and was psychometrically tested.

#### Participants, inclusion and exclusion criteria

The minimum age for study participants was set at 18 years. Participants had to be active in sports, and able to provide written informed consent. Only participants with German as their native language were allowed to participate (Table 1). All participants were informed about the rationale of the study. General exclusion criteria for all groups were pregnancy, low back pain, previous surgery of the lower extremity or spine, neurological, psychiatric, systemic diseases (e.g. rheumatic disease, connective tissue disease, diabetes mellitus), and current pension proceeding.

**Table 1:** Anthropometric characteristics and questionnaire results of participants, SF-12 (sub)scores (PCS and MCS) and OCLA (sub)scores (ADL and Sport). Age in years; height in cm; weight in kg; BMI in kg/m². SF-12 Physical and mental component score, given as deviation from the respective average score. OCLA-ADL and Sport Score in %. Between group differences for sex, age, height, weight, and BMI are p >0.05. SF-12 = Short-Form Health Survey

	OCLA (n = 40)	OFAC (n = 40)	SPORT AT RISK (n = 40)	LOW RISK SPORT (n = 40)
**SEX** (Male/Female)	22/18	18/22	20/20	24/16
**AGE** Mean (SD)	38.8 (14.1)	45.5 (14.9)	33.3 (13.5)	39.6 (15.7)
**HEIGHT** Mean (SD)	173.4 (10.3)	176.4 (9.1)	176.2 (9.9)	176.5 (8.8)
**WEIGHT** Mean (SD)	77.0 (18.0)	77.2 (17.0)	72.6 (13.6)	74.6 (11.4)
**BMI** Mean (SD)	25.5 (4.8)	24.6 (4.3)	23.2 (2.6)	23.9 (2.6)
**SF 12** Physical component score deviation from the respective average value (SD)	-6.2 (8.6)	-0.9 (8.5)	5.1 (4.6)	4.5 (5.0)
**SF-12** Mental component score deviation from the respective average value (SD)	1.6 (7.6)	2.7 (8.5)	3.4 (4.4)	3.6 (5.9)
**OCLA** ADL Score mean (SD)	34.8 (21.0)	33.3 (28.1)	3.3 (7.5)	1.9 (5.2)
**OCLA** Sport Score mean (SD)	60.7 (31.2)	43.6 (36.0)	2.2 (6.8)	4.3 (16.1)

OCLA = osteochondral lesion of the ankle; OFAC = other foot and ankle conditions; BMI = body mass index. SD = standard deviation.

The formula used to calculate the sample size was n = 16p(1 – p)/w^2^, where p is the expected ICC (selected ≥ 0.9) and w is the maximum width (0.20) of the 95% confidence interval [11]. Thus, the calculated minimum sample size for each group was 36 subjects. Four additional participants were included in each group to cover possible drop-outs. Nevertheless, the risk of bias checklist suggests a minimum sample size of 100 participants as a “rule of thumb” to meet the requirements of factor analysis, but the COSMIN group also acknowledges that a smaller sample might be appropriate [12, 13] and consensus was not reached on sample size requirements in a respective COSMIN Delphi study [14]. In addition, our study design called for completion after 3 years.

To test the psychometric properties of the OCLA-G questionnaire, 40 patients suffering from acute or chronic, symptomatic or asymptomatic OCLA were included in the “OCLA group”. Specific inclusion criteria for the OCLA group were: medically diagnosed osteochondral lesion (OCL) of the talus, distal tibial plafond, or malleoli, regardless of its origin (traumatic or nontraumatic) and regardless of previous treatment. Patients with bilateral OCLA, ankle fractures and recurrent ankle instability were excluded from the OCLA group. The OCLA patients were recruited through direct contact during the office hours in the orthopedic department of the Institute for Sports Medicine, Frankfurt am Main/Germany and the Department for Orthopedics and Trauma Surgery, University Hospital Freiburg/Germany. The recruitment of individuals from different centers and cities was used to minimize bias potentially related to specific patient selection or demographic factors. Patients in the OCLA group suffered 9.7 ± 18.6 months (range 1 day to 60.8 months) of the symptoms. Two (5%) patients had MRI grade I, two (5%) had grade II, 28 (70%) had grade III, and 6 (15%) had grade IV. For two (5%) patients, no respective information was available [15]. OCLA affected the medial talus in 32 (69.6%) patients, the lateral talus in six (13%), and the medial malleolus or lateral distal tibia in three (6.5%) patients each. The OCLA occurred at the lateral malleolus or at the central tibial plafond in one patient (2.2%) each. A triggering trauma to the ankle was reported by 46.2% of patients. Athletic activities were performed by 97.5% of the OCLA patients. The use of analgesics was necessary in 51.3% of the patients. The amount of exercise before the onset of symptoms was 3.2 ± 2.1 (range 1–10) h/week. The right or left ankle was affected in 44.7% and 55.3%, respectively. Pretreatment was surgical in 75% of patients.

In addition, three comparison groups were created (Table 1). To test whether the questionnaire is restrictive or exclusionary for patients and therefore might have diagnostic value for patients with OCLA, a second group of patients with other foot and ankle conditions (OFAC) was formed. The equality will show that the instrument is not suitable for diagnostic purposes. These patients were recruited through direct contact during the office hours in the orthopedic department of the Institute for Sports Medicine, Frankfurt am Main/Germany. The individuals in the OFAC group had injuries such as ankle sprains, recurrent ankle instability, tendinopathies, ankle fractures, or ankle arthritis that were diagnosed on clinical examination by a sports orthopaedic physician. Patients with OCLA and patients suffering from bipedal injuries were excluded from the OFAC group.

Forty uninjured persons playing sport at risk for the development of OCLA, but without any actual foot and ankle injuries or complaints within the past 1.5 years were included in the “sport at risk group”. These participants were recruited through direct contact during their training sessions in different sports clubs in the Rhein-Main area (Germany). In addition, participation of at least 1.5 h per week in a sport with OCLA risk (jogging, ball sports) was required. A further 40 people who played a sport with a low risk of developing OCLA (e.g. rowing, cycling) and had no actual foot or ankle injuries or complaints within the last 1.5 years formed the ‘low risk group’. Patients who regularly played ball sports were excluded from this group. These participants were also recruited through direct contact during training sessions in different clubs in the Rhein-Main area (Germany).

The anthropometric characteristics of the participants for the four study groups demonstrated no significant differences (Table 1).

### Phase 2: validation of the OCLA-G questionnaire (psychometric characteristics and statistical analyses)

The evaluation of the psychometric properties of the prefinal questionnaire included an analysis of its reliability (internal consistency and test-retest reliability), validity (face validity, known group validity, construct validity, concurrent validity and discriminant validity), and the assessment of floor and ceiling effects.

The data were processed in IBM SPSS® statistics version 27.0.1.0 64-Bit

#### Reliability

Internal consistency was calculated by Cronbach’s alpha values for both the ADL and sport subscores of the OCLA questionnaire. An acceptable to high level of internal consistency is associated with values between 0.70 and 0.95 [16].

In addition, corrected item-total scale statistics and Cronbach’s alpha values when a question was omitted were calculated by correlating the score of each item and the scale score minus the contribution of that item to the score. Values < 0.3 are considered insufficient [17].

To assess stability over time (test-retest reliability), the questionnaire was administered twice to 35 participants 2 to 16 days apart (mean, 4.2 days), including four OCLA patients, 10 OFAC patients, 13 sport at risk athletes, and eight low-risk individuals. Differences were compared for the OCLA-ADL and sport subscores using Spearman’s rank correlation coefficients for test-retest reliability. Spearman correlation coefficients were interpreted according to the literature: 0.0–0.20 = slight, 0.21–0.40 = fair, 0.41–0.60 = moderate, 0.61–0.80 = substantial and 0.81–1.0 = almost perfect agreement [18]. Only participants who described their condition as unchanged were included in the second survey.

#### Validity

The face validity [19] was assessed during the expert meetings by subjectively judging whether the items of the OCLA-G questionnaire captured the severity of a person’s OCLA-related symptoms. A symptomatic patient would thus have to achieve significantly higher values when answering the individual questions than, for example, a subject without ankle disease. In developing the questionnaire, consensus was reached by both patients and the two groups of experts during consensus discussions with respect to semantic, idiomatic, experiential, and conceptual issues. Four experts - a sports scientist, a physiotherapist and two orthopaedic surgeons with several years of experience in the management of sports injuries independently reviewed and rated the items of the first version of the questionnaire on a six-field Likert scale ranging from “very useful” to “not relevant”. Upon completion of their independent work, the experts convened to discuss the items until agreement was obtained.

“Known group validity as a form of external or construct validity is determined by the extent to which an instrument can demonstrate different scores for well-established distinct groups known to vary on the variables being measured [20].” Known group validity (= discriminating power) of the OCLA-G questionnaire was assessed by measuring the differences between the scores of the four participant groups [21]. Testing for differences in means between patients with an OCLS and the other groups was performed using the Games-Howell post hoc test. We respectively expected a difference in scores between the symptomatic and asymptomatic groups, but not between the OCLA and OFAC groups or between the asymptomatic high-risk and low-risk groups, as measured by the Games-Howell-Post-hoc-test for the ADL and sport subscores.

The concurrent validity correlates a measure with another that has been previously validated [21]. The OCLA-G questionnaire was tested against the SF-12 questionnaire [22]. We used version 2.0 of that generic instrument. It is divided into a physical and a mental score. The SF-12 consists of 12 questions and assesses “health-related quality of life. The SF-12 physical scale (PCS-12) score represents general health perception, physical functioning, physical role functioning, and pain. The mental health score (MCS-12) reflects emotional role functioning, mental well-being, negative affectivity, and social functioning.” [23, 24] Using available algorithms, the answers given can be transformed into a score for the so-called PCS-12 (Physical Component Scale) and the MCS-12 (Mental Component Scale) and the deviation from the respective average value can be determined. The more negative the deviation, the more symptomatic the respondents are. With respect to the OCLA-G questionnaire, we therefore expected a divergent correlation. As suggested in the literature [25], a linear relationship and a normal data distribution were established as prerequisites for the application of Pearson correlation (linear correlation coefficient). Pearson correlation analysis was then used to test the relationships between the individual sub-scores of the OCLA and the SF-12. Values of > 0.5 can be assumed to have a high, values of 0.3 to 0.5 a medium, and values < 0.3 a small correlation [26]. Floor and ceiling effects.

To sufficiently distinguish between patients with high and weak trait expressions, the questions were examined for floor and ceiling effects. The ceiling and floor effects indicate the ability of an instrument to assess the severity of a disease using an appropriate range of values. An instrument is considered to have ceiling and floor effects if more than 15% of the individuals completing the questionnaire score the maximum or minimum possible [16]. If ceiling effects are present, the question cannot sufficiently differentiate between patients with strong symptoms. In the case of floor effects, the questions cannot sufficiently differentiate between patients with weak symptoms [17].

#### Item analysis

In the exploratory data analysis, the item difficulty index for the OCLA patient group was calculated. A mean difficulty index between 20 and 80 was set as the target value. The purpose of the difficulty index is to distinguish between patients with high and low symptom expression. Therefore, items that are answered “yes” or “no” by all test persons do not provide much information [27]. The item difficulty (P_*i*_*)* was calculated: P_*i*_ = (item mean X_*i*_/maximum score) ×100.

The scores of the individual items were checked for normal distribution by calculation of skewness and kurtosis to detect conspicuous items to be removed from the score. If the skewness is > 0, the distribution is “right skewed”, the median is smaller than the arithmetic mean. If it is < 0, it is left-skewed, the median is greater than the arithmetic mean. A kurtosis of 0 corresponds to the curvature of a normal distribution. For values > 0, the test score distribution is more peaked, for values < 0, the distribution is flatter. Items with a kurtosis of +/- 7 and/or a skewness of +/- 2 and more or less are considered problematic [28].

The principal component analysis was performed for the OCLA patient group. To rule out whether a principal component analysis is reasonable, the Kaiser-Meyer-Olkin criterion (KMO) was determined. Values > 0.5 indicate that a principal component analysis is meaningful [29]. Subsequently, Bartlett’s test for sphericity was used to demonstrate the relationship between the individual questions. With values of p < 0.05, a relationship between the questions can be assumed, so that a principal component analysis is reasonable. The subsequent principal component analysis was used to show what proportion of the variance is explained by the respective component. Components explaining more than 10% of the total variance or whose eigenvalue was greater than or equal to 1 were considered [30, 31]. The number of factors and the eigenvalues were additionally displayed in a screeplot [31]. The factors determined from the principal component analysis and the screeplot were subjected to a rotation in which the eigenvalues were not changed [32]. The Oblimin rotation method with Kaiser normalization was chosen, in which oblique rotation is assumed and correlation of the factors is accepted and 0.4 was chosen as the limiting value of the loading [32].

The mean values of the ADL and the sport subscore of the OCLA-G questionnaire were evaluated separately. The F-test for heteroscedasticity was performed with the assumption that the variances of the mean values between the four groups differ. The assignability of the groups was illustrated by the test for between-subjects effects. The partial eta square was calculated to indicate what percentage of the variance could be explained by group membership. Therefore, in the absence of variance homogeneity, the Welch test was performed as a robust procedure to test for equality of means.

To specifically analyse the OCLA group, the collected scores were first tested for linearity and normal distribution. The linearity between the mean values was visualized graphically. In Q-Q plots the observed and the expected values were checked for normal distribution [33]. If normally distributed, Pearson correlations between the mean OCLA-G questionnaire scores and the deviations of the mean values of the PCS-12 and MSC-12 was calculated.

Where appropriate, the level of significance was set at p < 0.05.

## Results

### Phase 1: development of the questionnaire

The V1 version of the questionnaire consisted of 21 items. The three items with the lowest total column scores in the Likert-box assessment were excluded. According to the written comments of all experts (EG 1 + 2), one item was identified as unclear because it asked about pain during running or rotational movements. This item was rephrased to ask only about pain during running, while rotational movements were addressed in another item. Two other items were synthesized because both asked about symptoms during running. Respectively, the V2 version of the questionnaire consisted of 17 items.

The ten OCLA patients did not report any incomprehensibility or problems in answering or handling the V2 version of the questionnaire. Based on the written information and explanations, all patients were able to complete the questionnaire independently. The patients judged the readability and comprehensibility to be sufficient. Four and two questions, respectively, of the Sport subscale were not answered by two patients because they were not able to perform the required movements sufficiently. Following V2 testing two item were removed due to their low difficulty index (Pi = 2 and 12). Another item was deleted, although it achieved a difficulty index comparable to other questions, but was not considered by the experts to be additionally informative. Respectively, the V3 version of the questionnaire consisted of 14 items. This version 3 underwent further formal validation. Participants required 10–15 min to complete the questionnaire.

Further validation of the V3 version of the OCLA-G questionnaire (Phase 2) revealed that one item was poorly correlated with all other questions (Pearson‘s r = -0.316 to 0.286) and was therefore removed. Thus, the final OCLA-G questionnaire consists of 13 questions, divided in an ADL (8 items) and a sport subscale (5 items; Table 2).

**Table 2:** The OCLA-G (Osteochondral lesion of the ankle, German version) questionnaire. Questions 1–8 = ADL subscore, questions 9–13 = Sport subscore. The questions (Q) refer to the previous week and to the injured ankle

OCLA-G QUESTIONNAIRE. **Activity of daily life subscore** Q 1. How often have you had pain? Q 2. What is the maximum intensity of pain you have experienced? Q 3. How much did pain affect you at rest or at night? Q 4. How much were you affected by start-up pain (pain e.g. after prolonged sitting or in the morning after getting up)? Q 5. How much were you affected by pain at the end of the day? Q 6. How much were you affected by pain when walking on uneven ground? Q 7. How often have you had the feeling of locking/entrapement? Q 8. How often have you noticed swelling/effusion? **Sport subscore** Q 9. Did you feel any problems (such as pain, locking, feelings of instability) while running? Q 10. Did you feel any problems (such as pain, locking, feelings of instability) when bouncing, jumping? Q 11. Did you feel any problems (such as pain, locking, feelings of instability) when cutting or rotating? Q 12. How often did you have to discontinue your practiced sport? Q 13. How would you assess the current functionality of your ankle?	

### Phase 2: validation of the OCLA-G questionnaire

#### Reliability

Cronbach’s alpha values of the ADL (item 1–8) and sport (item 9–13) subscore were 0.950 and 0.965, respectively. The corrected item-total scale correlation of the ADL (item 1–8) and sport (item 9–13) subscore was 0.652–0.903 and 0.849–0.936, respectively. Cronbach’s alpha values when an item was omitted were 0.938–0.954 for the ADL (item 1–8) and 0.951–0.965 for the sport (item 9–13) subscore, respectively. Spearman’s rank correlation coefficients for test-retest reliability were 0.992 for the ADL subscale and 0.999 for the sport subscale, respectively (both p < 0.001).

#### Face validity

The face validity of the OCLA-G questionnaire was judged as good by the expert committee, as OCLA-related symptoms and severity were adequately represented. Following the independent evaluation of the 21 questions in the first version of the questionnaire, the experts agreed to delete three of the low-scoring questions. Two further questions were merged, while one item was further differentiated through discussion. This resulted in a second version of the questionnaire containing 17 questions.

#### Known group validity

The Games-Howell post-hoc test for the ADL and sport subscore demonstrated significantly higher scores for OCLA and OFAC patients compared to control participants (all p < 0.001). No significant difference was found between OCLA and OFAC patients and between the active healthy control subgroups (Table 3 + 4).

**Table 3:** Games-Howell post-hoc test for differences in mean OCLA-G scores between OCLA patients and the other subgroups for the ADL subscore. OCLA-G ADL = Osteochondral lesion of the ankle questionnaire, German version. OCLA = osteochondral lesion of the ankle. OFAC = other foot and ankle conditions

OCLA-G ADL			Mean difference	Standard error	p-value	95% Confidence interval	
						Lower limit	Upper limit
OCLA patients	vs.	OFAC patients	0.07500	0.27746	0.993	-0.6547	0.8047
		Sport at risk persons	1.57650	0.17628	**< 0.001**	1.1077	2.0453
		low risk persons	1.64550	0.17081	**< 0.001**	1.1893	2.1017

**Table 4:** Games-Howell post-hoc test for differences in mean OCLA-G scores between OCLA patients and the other subgroups for the sport subscore. OCLA-G = Osteochondral lesion of the ankle questionnaire, German version. OCLA = osteochondral lesion of the ankle. OFAC = other foot and ankle conditions

OCLA-G Sport			Mean difference	Standard error	p-value	95% Confidence interval	
						Lower limit	Upper limit
OCLA patients	vs.	OFAC patients	0.85139	0.41404	0.179	-0.2408	1.9436
		Sport at risk persons	2.92281	0.29033	**< 0.001**	2.1350	3.7106
		low risk persons	2.94103	0.28671	**< 0.001**	2.1609	3.7212

#### Concurrent validity

The subscores of the OCLA-G questionnaire were normally distributed and linear. There was a significant, high positive Pearson correlation between the ADL and sport subscore of the OCLA-G questionnaire (r = 0.853; p < 0.001; N = 143; Table 5). Compared to the mean deviation of the PCS-12 the Pearson correlation of the OCLA-G ADL and sport subscore was strong and divergent (r = -0.663 and − 0.647; both p < 0.001; N = 160 and 143; Table 5). Compared to the mean deviation of the MCS-12 the Pearson correlation of the OCLA-G ADL and sport subscore was small to medium and significantly divergent (r = -0.249 and − 0.164; p = 0.001 and 0.05; N = 160 and 143; Table 5). No significant correlation was found between the mean deviations of the PCS and the MCS subscores of the SF-12 questionnaire (r = 0.122; p = 0.124; N = 160; Table 5).

**Table 5:** Reliability and validity parameters of the OCLA-G subscores. * = p ≤ 0.05. ** = p ≤ 0.001. OCLA-G = Osteochondral lesion of the ankle questionnaire, German version. OCLA = osteochondral lesion of the ankle. OFAC = other foot and ankle conditions. ADL = activities of daily living. PCS-12 = physical component summary of Short-Form Health Survey. MCS-12 = mental component summary of Short-Form Health Survey

		OCLA-G	
		ADL	Sport
Reliability	Cronbach’s alpha	0.950	0.965
	Corrected item-total scale correlation	0.652–0.903	0.849–0.936
	Cronbach’s alpha when an item was omitted	0.938–0.954	0.951–0.965
Known group validity (p-values)	OCLA vs. OFAC	0.993	0.179
	OCLA vs. sport at risk	< 0.001	< 0.001
	OCLA vs. low risk	< 0.001	< 0.001
	OFAC vs. sport at risk	< 0.001	< 0.001
	OFAC vs. low risk	< 0.001	< 0.001
	Sport at risk vs. low risk	0.776	0.991
Concurrent validity (Pearson’s r)	ADL		0.853**
	PCS-12	-0.663**	-0.647**
	MCS-12	-0.249**	-0.164*

#### Construct validity

**Fig. 2:**
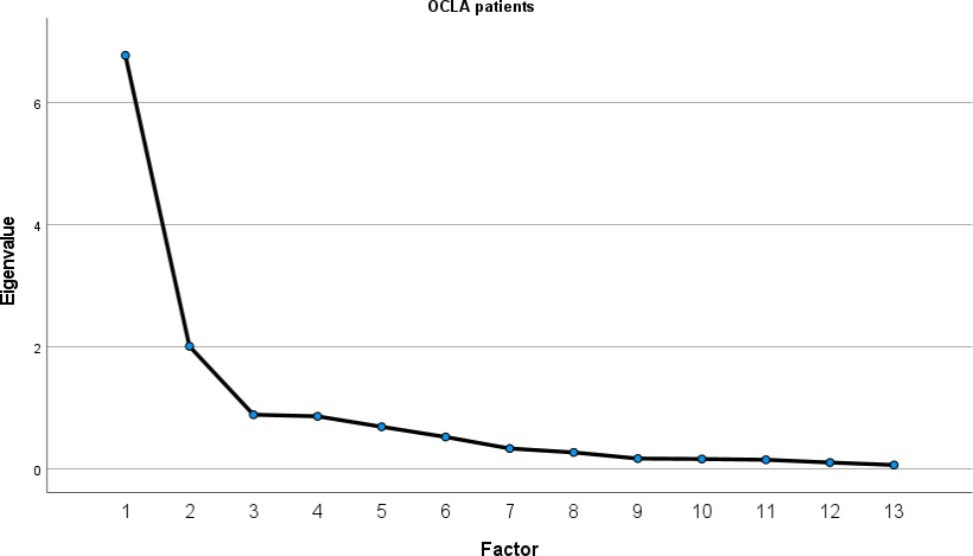
Screeplot to visualize the eigenvalues of the OCLA-G questionnaire. The screeplot shows a clear decrease of the eigenvalues. The eigenvalues of the first two factors are > 1, while the remaining factors are relatively small and hardly distinguishable from each other

The prerequisites that must be verified for the correct use of an exploratory factor analysis were satisfactorily met by a high Kaiser-Meyer-Olkin coefficient value (0.932) and a high significance on Bartlett’s test of sphericity (Chi2 = 2480.914; p < 0.001). To determine the number of factors to be extracted, an eigenvalue greater than one was specified [31], which was true for components 1 and 2. Principal component analysis for the OCLA group showed that 52.1% of the variance was represented in component 1 and 15.5% in component 2 (Fig. 2).

#### Floor and ceiling effects

No ceiling and floor effects were observed. Only four OCLA patients (10%) achieved the maximum score, while none of the participants achieved the minimum score of the possible range of values.

## Discussion

In the present study, a patient-oriented and disease-specific questionnaire for German-speaking patients with an osteochondral lesion of the ankle (OCLA-G questionnaire) was developed, validated, and tested for reliability. Previously, 46 different questionnaires were used to document the severity of OCLA symptoms but the OCLA-G questionnaire is the first validated instrument to measures the severity of symptoms, patient satisfaction, and the extent of functional limitations of OCLA patients in daily life and sports [3]. Analogue to different, multilingual translated, and internationally accepted region and/or disease specific questionnaires [34, 35], we divided the OCLA-G questionnaire into two sections. The ADL and the sport subscore assess the limitation of the patients at different levels of exertion. The sport subscore evaluates higher functional demands. The results are given in percentages (from 0 to 100) and separately for each subscore. A higher score represents a pronounced symptomatology. Since pain and dysfunction are often evident in OCLA, especially during sports, the sport subscore prevents a ceiling effect. Respectively, in the principal component analysis component 1 (items 1–8) showed a high correlation to the questions of the ADL subscore, while component 2 (items 9–13) correlated to the questions of the sport subscore.

The generation and validation of the OCLA-G questionnaire followed a standardized, rigorous and stepwise procedure as suggested in the literature: Item search, item reduction, questionnaire construction, final item reduction, and testing for validity and reliability [8–10]. Initially, 21 Items were generated. Following three steps of pretesting, 13 items remained for the final version.

The reliability of the OCLA-G questionnaire, examined by Cronbach’s alpha value, showed excellent internal consistency for ADL subscore (α = 0.95). However, according to Terwee et al. [16], internal consistency for the sport subdomain (α = 0.965) was not excellent, as values above 0.95 may indicate item redundancy in a given domain. Therefore, it is necessary to continue the research and analyses related to internal consistency, to determine whether or not the sport subscore contains redundant item(s). Test-retest reliability was ‘almost perfect’. Exploratory data analysis demonstrated moderate item difficulty. Face validity was good. The expert committee developed the items of the OCLA-G questionnaire through a structured content-analytic method. Known group validity demonstrated the expected differences between the distinct subgroups that were expected to score differently (patients vs. uninjured participants) but not between uninjured groups (at risk vs. low risk) and not between OCLA and OFAC patients. The fact that scores were not statistically different between the OCLA and OFAC groups indicates that the instrument is not limiting or exclusionary for patients with OCLA. Moreover, this result shows that the instrument is not suitable for diagnostic purposes. Testing the simultaneous validity of the ADL and sport subscore of the OCLA-G questionnaire yielded the expected positive correlation. Concurrent validity was tested against the SF-12 subscales. While the OCLA-G subscores were strongly correlated to the physical components (PCS-12), the correlation to the mental components (MCS-12) was only small to medium. This behavior can be explained by the fact that condition-specific PROMs provide advantages over generic tools. We expected a priori that OCLA would affect mental health less than physical health. Floor and ceiling effects were not observed.

Today, PROMs are the most recognized form of obtaining data on patient’s perspective of their health status and are increasingly used also in sports and exercise medicine [2]. They offer not only “a view of disease as a strictly biological phenomenon”, but provide “psychosocial consequences and functional impact” that “are most relevant to patients and therefore are key components in an assessment of the effect of disease or injury on health” [36]. The obtained data can be used in clinical practice, clinical research, and healthcare policy. OCLA is still a challenging issue for the surgeon and there is a paucity of clinical literature available. In addition to conservative treatment, various surgical interventions have been developed in recent years and “systematic reviews will be required to identify the optimal form of treatment for these lesions” [1, 37]. An initial literature search showed that 46 different instruments have previously been used to measure OCLA symptoms. However, among them no disease specific and validated PROM was found [3]. Therefore, the results of national and international research cannot yet be compared, and systematic reviews will not provide clinically relevant information. This gap can be filled by the OCLA questionnaire.

In our study sample, male participants (55%) slightly outnumbered female participants (45%; p = 0.636), while in the literature a male preponderance of 70% is described [1]. The participants were recruited from different cities in south-west Germany (Rhein-Main region and Freiburg i. Brsg.). This sampling method minimized the risk of bias associated with cultural, semantic, or demographic factors. There was no difference between the compared groups in terms of age. However, the mean age of the participants in this study (39.3 ± 15.1 years) was higher than the one reported in the literature (average age = 20–30 years) [1]. This inconsistency can be explained by the fact that we excluded participants under the age of 18.

The questionnaire is not intended to be used as a diagnostic tool but to record the degree of patient satisfaction and pain-related functional limitations in daily life and sports. Therefore, a thorough history taking and diagnostic assessment are still required before administering the OCLA-G questionnaire. Management strategies and decision-making are independent from the questionnaire results.

### Limitations

The primary limitations that should be considered in the present study include the small number of patients with OCLA. Specifically, results of factor analyses in our study are therefore not robust [12–14]. Responsiveness as a measurement over time analyses have not been performed due to the cross-sectional study design and has to be evaluated in further investigations. The OCLA-G was developed for German speaking patients. Therefore, no international comparisons can be made based on this instrument until further cross-cultural adaptations and validations are available in different languages, especially in English. Responsiveness and minimal clinically important difference (MCID) were not tested due to the study’s cross-sectional design but should be evaluated in further research. We have simultaneously developed an OCLA-G questionnaire for patients under 18 years of age, but this instrument has not yet been validated.

Another limitation relates to internal consistency for the sport subdomain (α = 0.965). As values above 0.95 may indicate item redundancy in a given domain [16] further internal consistency research is necessary to clarify that issue.

“Although the exact pathophysiology of the condition has not been clearly established, it is likely that a variety of etiological factors play a role, with trauma, typically from ankle sprains, being the most common” [1]. Our OCLA group included both, patients with traumatic and patients with non-traumatic history. The number of cases (n = 40), however, was too low for a subgroup analysis. A separate validity testing for chronic (osteochondritis dissecans) and traumatic (osteochondral fractures) lesions is therefore suggested. Accordingly, the different localizations of OCLA (medial talus, lateral talus, distal tibial plafond, medial malleolus, lateral malleolus) might influence the OCLA-G score and should be further comparatively investigated.

## Conclusions

The OCLA-G questionnaire was successfully developed, validated and tested for reliability for patients over 18 years of age. This PROM assesses the subjectively perceived symptoms (pain and function) of an OCLA patient. Further research is recommended to translate and validate this instrument in different languages. Additional clinimetric properties (responsiveness, accuracy, MCID) need to be assessed. Thus, international comparisons and a systematic review with meaningful numbers will allow to draw clinically relevant conclusions.

## Data Availability

The datasets used and analysed during the current study are available from the corresponding author on reasonable request.

## List of Abbreviations

ADL: Activities of Daily Living
DGOU: Deutsche Gesellschaft für Orthopädie und Unfallchirurgie
MCID: Minimal clinically important difference
MCS-12: Mental Component Summary of the SF-12
OCL: Osteochondral lesion
OCLA: Osteochondral lesion of the ankle
OCLA-G: Osteochondral lesion of the ankle, German version
OFAC: Other foot and ankle conditions
PCS-12: Physical Component Summary of the SF-12
PROMs: Patient reported outcome measures
SF-12: Short Form Health Survey 12
VISA: Victorian Institute of Sports Assessment
